# Differences in cancer pain management between outpatient and inpatient settings: A cross-sectional survey of nursing practices in China

**DOI:** 10.1007/s00520-025-10130-7

**Published:** 2025-11-18

**Authors:** Yingtao Meng, Jiao Lv, Yuanyuan Li, Xiao Han, Yali Wang

**Affiliations:** 1 Nursing Department, Shandong Cancer Hospital and Institute, Shandong First Medical University and Shandong Academy of Medical Sciences, Jinan, Shandong 250117 China; 2 Department of Gynecologic Oncology, Shandong Cancer Hospital and Institute, Shandong First Medical University and Shandong Academy of Medical Sciences, Jinan, Shandong 250117 China; 3 Department of Radiotherapy, Shandong Cancer Hospital and Institute, Shandong First Medical University and Shandong Academy of Medical Sciences, Jinan, Shandong 250117 China; 4 Department of Oncology, Shandong Cancer Hospital and Institute, Shandong First Medical University and Shandong Academy of Medical Sciences, Jinan, Shandong 250117 China

**Keywords:** Cancer pain, Pain management, Inpatient care, Outpatient care, Nursing practice, Opioid use

## Abstract

**Background:**

Effective cancer pain management is critical for patient quality of life but may vary significantly between inpatient and outpatient care settings. This study aimed to identify and compare pain assessment practices, patient behaviors, and treatment barriers across these environments.

**Methods:**

A cross-sectional survey was conducted among 730 nursing professionals from 75 hospitals across China. Respondents completed structured questionnaires evaluating pain assessment frequency, methods, treatment responses, and perceived barriers in both inpatient and outpatient settings. Comparative analyses were performed using chi-square tests or Fisher’s exact test. Multivariable adjusted logistic regression models were used to explore the associations.

**Results:**

Pain assessment was performed more frequently in inpatient settings (79.3%) than outpatient settings (57.6%) (difference, 21.8%; 95% CI, 4.6–38.9%; *P* < 0.01). Alignment with patients’ self-assessments did not differ significantly (71.9% inpatient vs. 63.6% outpatient; *P* > 0.05). Patients in inpatient care more often reported pain proactively (60.0% vs. 24.2%; difference, 35.7%; 95% CI: 20.7–50.8%; *P* < 0.01). Major outpatient barriers included difficulty in patient pain description (90.9% vs. 20.9%; difference, 70%; 95% CI, 59.7–80.2%; *P* < 0.01), while inpatient settings were affected by provider inattention to pain (82.4% vs. 21.2%; difference, 61.1%; 95% CI, 46.9–75.4%; *P* < 0.01). The proportion of patients receiving treatment after pain assessment was similar (74.9% inpatient vs. 66.7% outpatient; *P* > 0.05). However, outpatient nurses reported greater reluctance to prescribe opioids (24.2% vs. 7.0%; difference, 17.2%; 95% CI, 2.5–32.0%; *P* < 0.01), and outpatient settings saw a higher incidence of ineffective analgesia (33.3% vs. 18.8%; difference, 14.5%; 95% CI, -1.8–30.9%; *P* < 0.05).

**Conclusion:**

The exploratory cross-sectional survey suggests that cancer pain managements between outpatient and inpatient settings appear to be different, particularly in assessment frequency, patient communication, and opioid prescribing practices. Targeted, setting-specific interventions may be beneficial to optimize pain control and improve patient outcomes in both environments.

**Supplementary Information:**

The online version contains supplementary material available at 10.1007/s00520-025-10130-7.

## Introduction

Cancer is one of the leading causes of death in the world [1]. There were an estimated 19.96 million new cases and 9.74 million cancer-related deaths globally in 2022 [2]. Since 2000, both cancer incidence and mortality have risen steadily in China [3–5]. It has been suggested that 88% of cancer patients experience moderate to severe pain, with a median duration of six months [6]. Cancer pain severely impacts quality of life, affecting sleep, mobility, mood, daily activities, and interpersonal relationships [7, 8], and may lead to anxiety and depression [9].

The existing management approaches for cancer-related pain includes pharmacotherapy of opioids and other analgesics [10], which are the mainstay of cancer pain treatment but are not recommended for chronic pain in cancer survivors, as they are neither effective nor safe for long-term use. Apart from pharmacotherapy, it is empirically proven that non-pharmacological cognitive behavioral therapy (CBT) is a feasible and acceptable method to support cancer pain [11, 12] and is now available as an app [13]. Inadequate cancer pain assessment remains a major barrier to effective management [14]. For instance, 65.3% of physicians in Israel cited poor assessment as an obstacle [15].

Barriers to pain control involve patient-, provider-, and system-level factors. A cross-sectional survey in Chinese tertiary hospitals found that 54.52% of healthcare professionals believed patients were reluctant to report pain, and 58.1% felt patients described pain inaccurately [16]. This indicates that there are still numerous barriers to effective cancer pain management, such as proactive pain reporting frequency and patients’ knowledge/ability of accurately describing their pain. In outpatient settings, due to limited physician–patient interaction time and the busy schedules of physicians, cancer pain management differs significantly from that in inpatient settings. He-Ling Shi et al. reported that after standardized cancer pain treatment in outpatient settings, the proportion of patients with moderate to severe pain significantly decreased compared to before treatment (31.5% vs. 55.5%) [17]. Insufficient professional knowledge also contributes to poor pain management [18].

Previous investigations have largely concentrated on the comprehensive management of cancer pain within homogeneous settings, largely overlooking the nuances between outpatient and inpatient care [19–21]. Although the Chinese expert consensus on the assessment of cancer-related pain (2023 version) provides standardized guidelines [22], it does not differentiate between settings. Since the 2011 “Demonstration Wards for Standardized Cancer Pain Management” initiative, inpatient management has improved through training, standardized processes, and better follow-up [23]. However, only 15.4% of Chinese hospitals have dedicated nurses for outpatient cancer pain screening, far fewer than for inpatients [24].

To further address this gap between inpatient and outpatient settings, a disparity rooted in mechanisms such as workflow constraints, prescribing policies, and communication differences, we conducted a detailed survey among nursing staff from various hospitals across different cities in Shandong Province of China. This study compares pain assessment, management practices, and implementation barriers between outpatient and inpatient settings, aiming to identify distinct challenges and opportunities to improve cancer pain management.

## Methods

### Clinical data collection

An anonymous online survey was distributed to nursing staff who look after cancer patients experiencing pain across 75 hospitals in Shandong Province, including 58 tertiary hospitals and 17 secondary hospitals.

### Survey development

The survey instrument was developed through a structured process, informed by an extensive review of the literature on barriers to cancer pain management and in alignment with international guidelines, including the 2025 National Comprehensive Cancer Network (NCCN) Adult Cancer Pain Guidelines​. A multidisciplinary expert panel—comprising senior specialists in medical oncology, surgical oncology, radiology, pain medicine, and oncology nursing, each with more than ten years of clinical experience—collaboratively designed the questionnaire.

### Survey content

The finalized questionnaire was structured into four major domains:

1. Demographic and Institutional Characteristics:
  Included clinical experience, hospital classification (tertiary vs. secondary), department affiliation (oncology vs. non-oncology), presence of standardized cancer pain management wards, and whether pain assessments incurred additional charges.

2. Patient-Related Barriers:
  Explored patient cooperation, ability to describe pain accurately, and proactively reporting of pain.

3. Healthcare Provider-Related Barriers:
  Assessed engagement with pain assessment, methods used (e.g., verbal inquiry, structured scales such as the Numerical Rating Scale (NRS), Visual Analogue Scale (VAS), Verbal Rating Scale (VRS), Facial Expression Scale, Brief Pain Inventory (BPI), and the McGill Pain Questionnaire), and consistency between provider assessments and patients’ self-reports.

4. Healthcare System-Related Barriers:
  Investigated opioid prescribing restrictions, systemic implementation challenges, and barriers to translating assessments into treatment plans.

The questionnaire employed a mix of single-choice, multiple-choice, and five-point Likert scale items. A pilot test was conducted among 20 oncology nurses, and subsequent feedback informed minor revisions for improved clarity and comprehensiveness.

### Survey administration and data analysis

The finalized questionnaire (available as a supplementary document) was distributed via the Questionnaire Star online platform by trained staff to all eligible nurses (actively engaged in oncology care and had direct responsibilities in pain assessment or management for cancer patients from both inpatient units and outpatient oncology departments) through the organizational hierarchies of the 75 participating hospitals and was available for responses from March 15 to March 31, 2023. All responses were submitted anonymously. Prior to accessing the survey questions, an information sheet was presented, detailing the voluntary and anonymous nature of participation. Consent was implied upon voluntary completion and submission of the questionnaire. Upon closure of the survey, the organizing team compiled the submissions into a centralized database. Raw data were recorded using Microsoft Excel and analyzed with SPSS version 26.0. Descriptive statistics were used to summarize survey responses, and comparative analyses between inpatient (wards with overnight admission) and outpatient (clinics or day-treatment units without admission) settings were performed using the Chi-square test, or Fisher’s exact test, as appropriate. Mann–Whitney U test was used for ordinal data as a sensitivity analysis. Multivariable adjusted logistic regression with stepwise selection was performed to explore the associations. All such analyses were exploratory in nature and were not intended to confirm specific hypotheses.

## Results

### Participant demographics

A total of 759 questionnaires were distributed, of which 730 were valid, yielding a high response rate of 96.2%. Key demographics and institutional characteristics are presented in Table 1. Notably, a substantial sample size imbalance between the inpatient (*n* = 697) and outpatient (*n* = 33) groups was observed; given the small outpatient sample size, the finding should be interpreted as exploratory and requires validation in larger studies.

**Table 1:** Basic information of the survey data

Variable		Total	
		*N* = 730	Percentage
**Years of Experience**	< 2 years	267	36.58%
	2–5 years	223	30.55%
	> 5 years	240	32.88%
**Hospital Level**	Tertiary Hospital	640	87.67%
	Secondary Hospital	90	12.33%
**Department**	Oncology Department	608	83.29%
	Other Departments	122	16.71%
**Standardized Cancer Pain Treatment Demonstration Ward**	Yes	642	87.95%
	No	88	12.05%
**Assessment Fee**	Charged	600	82.19%
	Not Charged	130	17.81%

### Pain assessment frequency

Cancer pain assessments were significantly more frequent in inpatient settings compared to outpatient settings. Specifically, 79.3% of inpatient nurses reported assessing pain in over 91% of their patients, versus 57.6% in outpatient settings (difference, 21.8%; 95% CI, 4.6–38.9%; *P* < 0.01, Table 2). It should be noted that the wide 95% CIs observed for intergroup difference may indicate limited statistical power due to small sample sizes of inpatient subgroup. The difference was also observed in the sensitivity analysis of the ordinal assessment proportions (79.3% of inpatient setting vs. 57.6% of outpatient setting; *P* = 0.001; Supplementary Table 1). In univariable and multivariable analyses, inpatient setting, tertiary hospital setting, and charged assessment fee were all significant factors (*P* < 0.05) for the high proportion (> 91%) of cancer pain assessment, indicating they are independent influencing factors. After multivariable adjustment, high proportion (> 91%) of cancer pain assessment was more frequent in inpatient settings (OR, 2.83; 95% CI, 1.36–5.87; *P* < 0.01; Table 3).

**Table 2:** Comparison in inpatient and outpatient settings [cases (%)]

Comparison Items	Group	Outpatient	Inpatient	Difference^#^, % (95% CI)	χ^2^ Value	*P* Value
**Cancer Pain Assessment Rates**	Number of Cases	33	697	-	-	-
	> 91%	19(57.6)	553(79.3)	21.8 (4.6, 38.9)	8.800	0.003
	≤ 90%	14(42.4)	144(20.7)	-		
**Assessment Methods**	Number of Cases	33	697	-	-	-
	Questionnaire	23(69.7)	379(54.4)	−15.3 (−31.4, 0.8)	2.99	0.084
	Verbal Inquiry	31(93.9)	652(93.5)	−0.4 (−8.7, 8.0)	-	1.000^a^
	Other Methods	12(36.4)	24(3.4)	−32.9 (−49.4, −16.5)		< 0.001^a^
**Assessment Tools**	Number of Cases	33	697	-	-	-
	NRS	29(87.9)	651(93.4)	5.5 (−5.8, 16.8)	-	0.274^a^
	VRS	12(36.4)	221(31.7)	−4.7 (−21.4, 12.1)	0.314	0.575
	VAS	4(12.1)	155(22.2)	10.1 (−1.4, 21.7)	1.893	0.169
	Facial Expression Scale	26(78.8)	532(76.3)	−2.5 (−16.8, 11.8)	0.106	0.745
	BPI	4(12.1)	157(22.5)	10.4 (−1.2, 22.0)	1.984	0.159
	McGill Pain Questionnaire	1(3.0)	30(4.3)	1.3 (−4.8, 7.3)	-	1.000^a^
	Other	0(0.0)	4(0.6)	0.6 (0.01, 1.1)	-	1.000^a^
**Consistency Between Nurse Assessment and Patient Self-Assessment**	Number of Cases	33	697	-	-	-
	Consistent	21(63.6)	501(71.9)	-	2.344	0.310
	Rating Higher Than Patient's	7(21.2)	85(12.2)	-	-	-
	Rating Lower Than Patient's	5(15.2)	111(15.9)	-	-	-
**Proportion of Patients Proactively Reporting Pain**	Number of Cases	33	697	-	-	-
	> 91%	8(24.2)	418(60.0)	35.7 (20.7, 50.8)	16.551	< 0.001
	≤ 90%	25(75.8)	279(40.0)	-		
**Barriers to Assessment Implementation**	Number of Cases	33	697	-	-	-
	Patient's Willingness to Cooperate Insufficient	18(54.5)	377(54.1)	−0.5 (−17.8, 16.9)	0.003	0.959
	Patient Difficulty in Accurate Description	30(90.9)	146(20.9)	−70.0 (−80.2, −59.7)	84.290	< 0.001
	Lack of Attention from Healthcare Providers	7(21.2)	574(82.4)	61.1 (46.9, 75.4)	72.505	< 0.001
	Insufficient Communication and Feedback	13(39.4)	185(26.5)	−12.9 (−29.8, 4.1)	2.633	0.105
	Other	0(0.0)	7(1.0)	1.0 (0.3, 1.7)	-	1.000^a^
**Proportion of Pain Management Following Assessment**	Number of Cases	33	697			
	> 91%	22(66.7)	522(74.9)	8.2 (−8.2, 24.6)	1.123	0.289
	≤ 90%	11(33.3)	175(25.1)	-		
**Barriers to Pain Treatment Following Assessment**	Number of Cases	33	697	-	-	-
	Policy Impact on Analgesic Use	13(39.4)	184(26.4)	−13.0 (−30.0, 4.0)	2.700	0.100
	Patient Fear of Side Effects	29(87.9)	561(80.5)	−7.4 (−18.9, 4.1)	1.110	0.292
	Patient Fear of Opioid Addiction	20(60.6)	493(70.7)	10.1 (−6.9, 27.1)	1.546	0.214
	Physician Reluctance to Prescribe Opioids	8(24.2)	49(7.0)	−17.2 (−32.0, −2.5)	-	0.0025^a^
	Insufficient Efficacy of Prescribed Analgesics	11(33.3)	131(18.8)	−14.5 (−30.9, 1.8)	4.251	0.039
	Other	0(0.0)	17(2.4)	2.4 (1.3, 3.6)	-	1.000^a^

^#^The proportion of inpatient group minus the proportion of outpatient group

^a^Fisher’s exact test

**Table 3:** Factors associated with cancer pain management

	Univariable logistic regression		Multivariable logistic regression	
	OR (95% CI)	*P* value	OR (95% CI)	*P* value
**Cancer Pain Assessment Rates, > 91%**				
Inpatient vs. outpatient	2.83(1.39,5.78)	0.004	2.83(1.36,5.87)	0.005
Tertiary hospital vs. secondary hospital	2.12(1.32,3.42)	0.002	2.14(1.32,3.47)	0.002
Assessment fee: charged vs. not charged	1.9(1.25,2.9)	0.003	1.7(1.1,2.61)	0.017
Oncology department vs. other departments	0.81(0.5,1.33)	0.413	-	-
Standardized cancer pain treatment demonstration ward (yes vs. no)	1.24(0.74,2.08)	0.416	-	-
**Proportion of Patients Proactively Reporting Pain, > 91%**				
Inpatient vs. outpatient	4.68(2.08,10.53)	< 0.001	3.85(1.69,8.81)	0.001
Tertiary hospital vs. secondary hospital	1.2(0.77,1.87)	0.422	-	-
Assessment fee: charged vs. not charged	1.63(1.11,2.38)	0.012	1.48(1,2.19)	0.050
Oncology department vs. other departments	1.5(1.02,2.22)	0.041	1.29(0.85,1.95)	0.235
Standardized cancer pain treatment demonstration ward (yes vs. no)	2.01(1.28,3.15)	0.002	1.58(0.97,2.56)	0.065
**Barriers to Pain Assessment- difficult to accurately describe their pain**				
Outpatient vs. inpatient	37.74(11.36,125.37)	< 0.001	37.74(11.36, 125.37)	< 0.001
Tertiary hospital vs. secondary hospital	1.42(0.81,2.48)	0.218	-	-
Assessment fee: charged vs. not charged	0.97(0.62,1.5)	0.881	-	-
Oncology department vs. other departments	0.87(0.56,1.36)	0.549	-	-
Standardized cancer pain treatment demonstration ward (yes vs. no)	0.83(0.5,1.37)	0.460	-	-
**Barriers to Pain Assessment- lack of attention from healthcare providers**				
Inpatient vs. outpatient	17.33(7.35,40.82)	< 0.001	25.75(9.86,67.25)	< 0.001
Tertiary hospital vs. secondary hospital	0.51(0.26,0.98)	0.043	0.55(0.28,1.08)	0.083
Assessment fee: charged vs. not charged	1.21(0.77,1.91)	0.406	-	-
Oncology department vs. other departments	0.63(0.37,1.08)	0.092	0.51(0.27,0.95)	0.033
Standardized cancer pain treatment demonstration ward (yes vs. no)	0.78(0.43,1.4)	0.405	0.47(0.22,1.04)	0.061
**Barriers to Pain Treatment- physician reluctance to prescribe opioids**				
Outpatient vs. inpatient	4.23(1.81,9.88)	0.001	4.23(1.81,9.88)	0.001
Tertiary hospital vs. secondary hospital	1(0.44,2.29)	0.991	-	-
Assessment fee: charged vs. not charged	1.02(0.5,2.08)	0.957	-	-
Oncology department vs. other departments	0.73(0.38,1.43)	0.361	-	-
Standardized cancer pain treatment demonstration ward (yes vs. no)	0.71(0.34,1.5)	0.369	-	-
**Barriers to Pain Treatment- ineffective analgesic outcomes**				
Outpatient vs. inpatient	2.16(1.02,4.57)	0.044	2.21(1.03,4.72)	0.041
Tertiary hospital vs. secondary hospital	1.66(0.88,3.13)	0.121	1.55(0.82,2.95)	0.179
Assessment fee: charged vs. not charged	1.31(0.79,2.18)	0.296	1.35(0.81,2.27)	0.254
Oncology department vs. other departments	0.77(0.48,1.24)	0.286	-	-
Standardized cancer pain treatment demonstration ward (yes vs. no)	0.93(0.54,1.62)	0.800	-	-

OR = odds ratio. Multivariable logistic regression with stepwise selection was used to calculate the ORs and their 95% CI and P values. Care settings (inpatient vs. outpatient), hospital level (tertiary hospital vs. secondary hospital), department (oncology department vs. other departments), standardized cancer pain treatment demonstration ward (yes vs. no) and assessment fee (charged vs. not charged) were initially considered for selection. *N* = 730 for all the regressions

### Assessment methods and tools

Verbal inquiry was the most common method for pain assessment in both settings (93.9% outpatient vs. 93.5% inpatient; *P* = 1.00). A non-significant trend was observed towards more frequent use of questionnaires in outpatient settings (69.7% vs. 54.4%, *P* = 0.08) (*P* = 0.08). Notably, alternative assessment methods were reported significantly more often in outpatient care (36.4% vs. 3.4%; difference, 32.9%; 95% CI, 16.5–49.4%; *P* < 0.001).

With regard to pain assessment tools, both inpatient and outpatient nurses predominantly used the Numerical Rating Scale (NRS) and the Facial Expression Scale, with no significant differences observed between the two groups (NRS: 93.4% inpatient vs. 87.9% outpatient; *P* = 0.274; Facial Expression Scale: 76.3% vs. 78.8%; *P* = 0.75). No statistically significant differences were observed in the usage rates for other tools such as the Verbal Rating Scale (VRS) (31.7% vs. 36.4%; difference, −4.7%; 95% CI, −21.4–12.1%), Visual Analogue Scale (VAS) (22.2% vs. 12.1%; difference, 10.1%; 95% CI, −1.4–21.7%), Brief Pain Inventory (BPI) (22.5% vs. 12.1%; difference, 10.4%; 95% CI, −1.2–22.0%), and McGill Pain Questionnaire (4.3% vs. 3%; difference, 1.3%; 95% CI, −4.8–7.3%) across inpatient and outpatient settings (all *P* > 0.05, Table 2).

### Concordance with patient self-assessments

Most nurses reported that their assessments were consistent with patients' self-assessments based on pain severity scores, with a smaller proportion indicating that their ratings were either higher or lower than those of the patients. There were no significant differences between outpatient and inpatient settings in these proportions (*P* > 0.05) (Table 2).

### Patient-initiated pain reporting

Inpatient settings demonstrated a significantly higher proportion of patients proactively reporting their pain (60.0%) compared to outpatient settings (24.2%) (difference, 35.7%; 95% CI, 20.7–50.8%; *P* < 0.001; Table 2). The results of the sensitivity analysis were consistent (*P* = 0.001, Supplementary Table 1). After multivariable adjustment, high proportion (> 91%) of patients proactively reporting pain was more frequent in inpatient settings, with odds increased by 285% in outpatient settings (OR, 3.85; 95% CI, 1.69–8.81; *P* < 0.01; Table 3). It is noteworthy that the factors significant in univariable analysis (assessment fee, department, ward) were no longer significant in multivariable analysis, suggesting confounding effects, while inpatient setting remained an independent influencing factor.

### Barriers to pain assessment

During the assessment process, a substantially higher proportion of patients in the outpatient setting had difficulty accurately describing their pain (90.9% vs. 20.9%; difference, 70.0%; 95% CI, 59.7–80.2%; *P* < 0.01; Table 2; OR, 37.74; 95% CI: 11.36–125.37; *P* < 0.01; Table 3), whereas other influencing factors did not show statistical significance in univariable analysis. A considerably greater lack of attention from healthcare providers was noted in the inpatient setting (82.4% vs. 21.2%; difference, 61.1%; 95% CI, 46.9–75.4%; *P* < 0.01, Table 2; OR, 25.75; 95% CI, 9.86–67.25; *P* < 0.01; Table 3). The wide confidence intervals of the odds ratio should be noted and may reflect the limited sample size of 33 in outpatient group. Additionally, tertiary hospital showed marginal significance in both univariable and multivariable analyses for the assessment of lack of attention from healthcare providers. Hypotheses for further investigation could be formulated based on the consistent direction of the effect along with a trend toward significance. There were no significant differences between the two settings regarding patients' willingness to cooperate or insufficient communication and feedback from healthcare providers (both *P* > 0.05) (Table 2).

### Pain management following assessment

The proportion of patients receiving pain management after assessment was slightly higher in inpatient settings (74.9%) compared to outpatient settings (66.7%), but the difference (8.2%; 95% CI, −8.2–24.6%) was not statistically significant (***P* = 0.29**).

### Barriers to pain treatment

Outpatient nurses were more likely to report physician reluctance to prescribe opioids (24.2% vs. 7.0%; difference, 17.2%; 95% CI, 2.5–32.0%; ***P* < 0.01**; Table 2; OR, 4.23; 95% CI: 1.81–9.88; *P* < 0.01; Table 3) and slightly more likely to note ineffective analgesic outcomes (33.3% vs. 18.8%; difference, 14.5%; 95% CI: −1.8–30.9%; ***P* = 0.039**; Table 2; OR, 2.21; 95% CI, 1.03–4.72; *P* = 0.041; Table 3). Similarly, these findings are considered as preliminary and exploratory due to the limited sample size of outpatient group. Other barriers, including policy constraints on analgesic use, patient fear of side effects, and concerns about opioid addiction, did not differ significantly between settings (all ***P* > 0.05**, Table 2).

## Discussion

In recent years, it has seen a growing emphasis on cancer pain assessment in clinical practice in China, driven by initiatives such as the creation of cancer pain demonstration wards and the introduction of cancer pain assessment fees. These policies have provided a solid theoretical foundation for healthcare professionals and enabled them to gain valuable practical experience in assessing pain and managing analgesic treatment. This study highlights substantial disparities in cancer pain management between outpatient and inpatient settings. Our findings reveal that inpatient settings have a significantly higher frequency of pain assessments, with assessments conducted in 79.3% of cases compared to only 57.6% in outpatient settings (*P* < 0.01). This discrepancy underlines a critical gap in the proactive management of cancer pain, exacerbated by operational challenges in each setting. The study underscores the necessity for customized strategies tailored to the unique challenges of each setting.

The assessment rate for cancer pain in outpatient settings is lower compared to inpatient settings. International studies have similarly found that only 77% (82/106) of patients underwent at least one pain screening within three months of their visit [25]. The 2025 National Comprehensive Cancer Network (NCCN) guidelines in the United States recommend that healthcare providers conduct pain screenings at every visit for cancer patients [26]. Pain assessment data should be incorporated into pain management decisions [27]. However, the actual rate of assessment falls short of the guideline's recommendation for evaluation at every visit. In outpatient settings, due to time constraints and the focus on tumor-related treatment plans, pain assessment is often overlooked. This oversight can result in undetected cancer pain or insufficient adjustment of analgesic dosages, leading to inadequate pain relief.

Inadequate Patient Education as a Barrier to Assessment and Management in Outpatient Settings. The study found that patients in outpatient settings were less likely to proactively report pain compared to those in inpatient settings. This finding is consistent with research by Kim et al. [28], which reported that 53.5% of patients were reluctant to report pain, 65% feared side effects, and 67.2% were concerned about addiction. Additionally, this study revealed that patients in outpatient settings had greater difficulty accurately describing their pain. These findings suggest significant gaps and misconceptions in patients' knowledge, attitudes, and coping strategies regarding pain. The 2025 NCCN Adult Cancer Pain Guidelines also emphasize the need to educate patients and their families/caregivers on various aspects, including providing educational materials, understanding the significance and consequences of pain, setting expectations/knowledge/treatment plans for pain management, and understanding the use and risks of opioids [26]. Enhancing patient education and fostering a rational understanding of medications can play a crucial role in the accurate and effective assessment and management of cancer pain. Besides inadequate patient education, other barriers to effective pain assessment and management in outpatient settings may include limited consultation time and insufficient communication between patients and healthcare providers.

The Impact of Healthcare Providers on Cancer Pain Management Differs Across Clinical Settings. Although inpatient settings demonstrate higher pain assessment rates, this likely reflects mandatory documentation requirement rather than strong clinician engagement. The persistence of inadequate pain attention, even where assessments are routinely conducted, suggests underlying issues such as insufficient training, clinical prioritization, or excessive workload that diminish the perceived importance of pain evaluation. Furthermore, the increased incidence of under-prescription of analgesics across both settings points to structural and regulatory barriers, including stringent opioid-prescribing policies, complex prescription processes, and persistent concerns about addiction and misuse. In particular, the tendency to prescribe weak opioids or non-opioid analgesics for moderate-to-severe pain may reflect system-level caution regarding controlled substances, even when stronger analgesics are clinically indicated [28]. This is consistent with earlier findings, including a 2016 Chinese national survey which showed that nearly 50% of cancer patients with moderate pain were still prescribed non-potent opioids and 25% of those assessed with severe pain had not been prescribed strong opioids. The observed reluctance among outpatient providers to initiate or escalate opioid therapy may also be influenced by contextual constraints such as brief consultation times, lack of follow-up mechanisms, and limited care coordination. By contrast, inpatient settings—though better equipped for monitoring—may still be affected by inflexible formularies, inconsistent adherence to guidelines, or interprofessional communication gaps. These findings highlight the need to move beyond workflow compliance and address the deeper organizational, educational, and regulatory determinants that shape prescribing behaviors and assessment practices across different clinical environments.

This study, conducted through a survey of healthcare providers in China, revealed variations in cancer pain management between outpatient and inpatient settings. Despite various external factors, the primary assessment methods and tools used in both environments suggested consistency, reflecting the persistent efforts of healthcare professionals to optimize cancer pain management. Therefore, it might be valuable to explore tailored management strategies with preliminary suggestions such as: enhancing patient education and increasing assessment rates in outpatient settings, improving communication between doctors and patients about cancer pain, and addressing barriers to prescribing opioids. In inpatient settings, future work might benefit from improving the subjective importance of cancer pain management and better optimization of analgesic choices.

The limitations of this study include the broad scope of the online survey, which resulted in significant variations in the understanding and practice of cancer pain assessment across different departments. Additionally, the relatively small sample size of outpatient pain nurses further constrains interpretability of the findings, given the pronounced imbalance between the outpatient (n = 33) and inpatient (n = 697) settings. Therefore, the comparisons should be considered as exploratory, and may reduce the external validity of any outpatient-specific conclusions. Moreover, utilizing a cluster-based approach, the survey link was distributed internally to ensure that all eligible nurses within these institutions had access to the Questionnaire Star platform. However, potential non-response bias remains a limitation, as factors such as high clinical workload could influence a nurse's decision or availability to participate, which may affect the generalizability of the findings. In addition, our sample was predominantly from tertiary hospitals (87.7%) and oncology departments (83.3%), both of which are factors that may confound the results by influencing pain management practices. Our current dataset does not permit a robust examination of their influence, given that the small cell sizes in the minority groups (e.g., secondary hospitals, non-oncology departments) would render any subgroup analyses underpowered and potentially misleading. Another important limitation is the reliance on self-reported data, which may be subject to social desirability and recall biases. Responses regarding practices and barriers reflect perceived rather than objectively measured behaviors, and may not always align with actual clinical performance or institutional protocols. Although self-report provides valuable insights into individual perspectives. Nonetheless, the overall patterns observed offer valuable preliminary insights that can help inform the design of future, larger-scale studies specifically aimed at comparing settings and exploring these issues in greater depth.

In conclusion, our findings suggest that addressing these identified discrepancies by implementing targeted interventions may help improve the quality of life for patients suffering from cancer pain. Outpatient settings may benefit from introducing nurse-led screening protocols or standardized pain documentation tools to improve assessment accuracy and consistency, while the implementation of daily pain management audit and feedback systems can be helpful in inpatient setting. For outpatient settings, enhancing patient education, increasing the frequency of pain assessments, and improving communication between patients and providers may also be considered.

## Supplementary Information

Below is the link to the electronic supplementary material.

Supplementary file1 (DOCX 18 KB)

Supplementary file2 (DOCX 5013 KB)

## Data Availability

The datasets used and analyzed during the current study are available from corresponding authors on reasonable request.
