# Supplementary material for: Differences in cancer pain management between outpatient and inpatient settings: A cross-sectional survey of nursing practices in China
Source: Support Care Cancer. 2025 Nov 18;33(12):1080. doi: 10.1007/s00520-025-10130-7 (PMC12627119; doi:10.1007/s00520-025-10130-7)
Supplement: Supplementary file 1 — Supplementary file1 (DOCX 18 KB) [file 520_2025_10130_MOESM1_ESM.docx]

**Supplementary table 1. Comparison in Inpatient and Outpatient Settings [Cases (%)]**

| **Comparison Items** | **Outpatient**  **(N=33)** | **Inpatient**  **(N=697)** | **\|Z\|^a^** | **P Value^a^** |
| --- | --- | --- | --- | --- |
| **Cancer Pain Assessment Rates** | | | | |
| ≤10% | 0(0.0) | 10(1.4) | 3.264 | 0.001 |
| 11%-25% | 1(3.0) | 17(2.4) |  |  |
| 26%-50% | 5(15.2) | 19(2.7) |  |  |
| 51%-75% | 7(21.2) | 28(4.0) |  |  |
| 76%-90% | 1(3.0) | 70(10.0) |  |  |
| >91% | 19(57.6) | 553(79.3) |  |  |
| **Proportion of Patients Proactively Reporting Pain** | | | | |
| ≤10% | 5(15.2) | 10(1.4) | 4.962 | <0.001 |
| 11%-25% | 3(9.1) | 27(3.9) |  |  |
| 26%-50% | 6(18.2) | 45(6.5) |  |  |
| 51%-75% | 6(18.2) | 93(13.3) |  |  |
| 76%-90% | 5(15.2) | 104(14.9) |  |  |
| >91% | 8(24.2) | 418(60.0) |  |  |
| **Proportion of Pain Management Following Assessment** | | | | |
| ≤10% | 0(0.0) | 3(0.4) | 1.443 | 0.149 |
| 11%-25% | 2(6.1) | 5(0.7) |  |  |
| 26%-50% | 4(12.1) | 29(4.2) |  |  |
| 51%-75% | 2(6.1) | 44(6.3) |  |  |
| 76%-90% | 3(9.1) | 94(13.5) |  |  |
| >91% | 22(66.7) | 522(74.9) |  |  |

^a^ Mann-Whitney U test.
