# Supplementary material for: Differences in cancer pain management between outpatient and inpatient settings: A cross-sectional survey of nursing practices in China
Source: Support Care Cancer. 2025 Nov 18;33(12):1080. doi: 10.1007/s00520-025-10130-7 (PMC12627119; doi:10.1007/s00520-025-10130-7)
Supplement: Supplementary file 2 — Supplementary file2 (DOCX 5013 KB) [file 520_2025_10130_MOESM2_ESM.docx]

**Shandong Province Cancer Pain Nursing Screening and Assessment Status Survey**

Dear Expert, thank you for participating in this survey. Please fill it out according to your actual situation.

1. Hospital Name

[Fill-in-the-blank]

_________________________________

2. City of the Hospital

[Single-choice question]

○ Jinan

○ Qingdao

○ Yantai

○ Weihai

○ Dongying

○ Zibo

○ Weifang

○ Rizhao

○ Laiwu

○ Heze

○ Zaozhuang

○ Dezhou

○ Binzhou

○ Linyi

○ Jining

○ Liaocheng

○ Tai'an

3. Hospital Level

[Single-choice question]

○ Tertiary general hospital

○ Tertiary oncology hospital

○ Secondary general hospital

○ Secondary oncology hospital

○ Other _________________

4. Your Department

[Single-choice question]

○ Oncology

○ Radiotherapy

○ Pain Management

○ Respiratory Medicine

○ Gastroenterology

○ Nephrology

○ Neurology

○ Gynecology

○ Endocrinology

○ Hematology

○ Other _________________

5. Does your department charge a fee for screening and assessment?

[Single-choice question]

○ Yes

○ No

6. Is your department a Standardized Cancer Pain Treatment Demonstration Ward?

[Single-choice question]

○ Yes

○ No

7. Your primary work environment is:

[Single-choice question]

○ Outpatient clinic

○ Inpatient ward

***Questions for Outpatient Clinic Staff (Dependent on question 7, option 1)***

8. Proportion of screening and assessment in the outpatient clinic:

[Single-choice question]

○ Below 10%

○ 11%-25%

○ 26%-50%

○ 51%-75%

○ 76%-90%

○ Above 91%

9. Proportion of outpatient patients who actively report pain:

[Single-choice question]

○ Below 10%

○ 11%-25%

○ 26%-50%

○ 51%-75%

○ 75%-90%

○ Above 91%

10. Who is responsible for screening and assessment in the outpatient clinic?

[Multiple-choice question]

□ Doctor

□ Nurse

□ Other _________________

11. Methods for screening and assessment in the outpatient clinic:

[Multiple-choice question]

□ Questionnaire survey

□ Oral inquiry

□ Other _________________

12. Tools for screening and assessment in the outpatient clinic:

[Multiple-choice question]

□ NRS (Numerical Rating Scale)

□ VRS (Verbal Rating Scale)

□ VAS (Visual Analog Scale)

□ Faces Pain Scale

□ BPI (Brief Pain Inventory)

□ McGill Pain Questionnaire

□ Other _________________

13. Obstacles encountered during the screening and assessment process in the outpatient clinic:

[Multiple-choice question]

□ Lack of patient willingness to cooperate

□ Difficulty for patients to accurately describe pain

□ Insufficient attention from medical staff

□ Insufficient communication and feedback between medical staff

□ Other _________________

14. What is the proportion of outpatient patients with identified pain who subsequently receive appropriate treatment?

[Single-choice question]

○ Below 10%

○ 11%-25%

○ 26%-50%

○ 51%-75%

○ 76%-90%

○ Above 91%

15. For outpatients with identified pain, what are the reasons for not receiving adequate subsequent treatment?

[Multiple-choice question]

□ Policies affecting the use of analgesic drugs

□ Patient fear of adverse reactions

□ Patient fear of opioid addiction

□ Doctor's reluctance to prescribe opioids

□ Insufficient analgesic efficacy of prescribed drugs

□ Other _________________

16. How do you think the pain scores from outpatient healthcare providers compare to the patients' own scores?

[Single-choice question]

○ Consistent

○ Higher than the patient's score

○ Lower than the patient's score

***Questions for Inpatient Ward Staff (Dependent on question 7, option 2)***

17. Proportion of screening and assessment in the inpatient ward:

[Single-choice question]

○ Below 10%

○ 11%-25%

○ 26%-50%

○ 51%-75%

○ 76%-90%

○ Above 91%

18. Proportion of inpatient patients who actively report pain:

[Single-choice question]

○ Below 10%

○ 11%-25%

○ 26%-50%

○ 51%-75%

○ 75%-90%

○ Above 91%

19. Methods for screening and assessment in the inpatient ward:

[Multiple-choice question]

□ Questionnaire survey

□ Oral inquiry

□ Other _________________

20. Tools for screening and assessment in the inpatient ward:

[Multiple-choice question]

□ NRS (Numerical Rating Scale)

□ VRS (Verbal Rating Scale)

□ VAS (Visual Analog Scale)

□ Faces Pain Scale

□ BPI (Brief Pain Inventory)

□ McGill Pain Questionnaire

□ Other _________________

21. What are the biggest obstacles encountered during the screening and assessment process in the inpatient ward?

[Multiple-choice question]

□ Lack of patient willingness to cooperate

□ Insufficient attention from medical staff

□ Difficulty for patients to accurately describe pain

□ Insufficient communication and feedback between medical staff

□ Other _________________

22. What is the proportion of inpatient patients with identified pain who subsequently receive appropriate treatment?

[Single-choice question]

○ Below 10%

○ 11%-25%

○ 26%-50%

○ 51%-75%

○ 76%-90%

○ Above 91%

23. For inpatients with identified pain, what are the reasons for not receiving adequate subsequent treatment?

[Multiple-choice question]

□ Policies affecting the use of analgesic drugs

□ Patient fear of adverse reactions

□ Patient fear of opioid addiction

□ Doctor's reluctance to prescribe opioids

□ Insufficient analgesic efficacy of prescribed drugs

□ Other _________________

24. How do you think the pain scores from inpatient healthcare providers compare to the patients' own scores?

[Single-choice question]

○ Consistent

○ Higher than the patient's score

○ Lower than the patient's score

25. How frequently do you assess pain in inpatient patients after they have taken analgesics?

[Single-choice question]

○ ≥ 2 times a day

○ Once a day

○ Once every two days

○ Once every 3 days or more

***Questions about Screening/Assessment Fees***

26. What do you think are the reasons your department has not implemented a fee for screening and assessment?

[Multiple-choice question]

*(Dependent on question 5, option 2 "No")*

□ Hospital policy reasons

□ Departmental mindset reasons

□ Insufficient nursing skills and knowledge

□ Lack of confidence in nursing

□ Other _________________

27. In which aspects of cancer pain management do you think charging a fee for screening and assessment could lead to improvement?

[Multiple-choice question]

*(Dependent on question 5, option 2 "No")*

□ Baseline pain

□ Breakthrough pain

□ Titration

□ Dose adjustment after pain changes

□ Follow-up

□ Patient education

□ Other _________________

28. When did the implementation of the screening and assessment fee begin?

[Single-choice question]

*(Dependent on question 5, option 1 "Yes")*

○ 2016-2017

○ 2018-2019

○ 2020-2021

○ 2022-2023

29. After the implementation of the screening and assessment fee, which of the following aspects do you think have improved?

[Multiple-choice question]

*(Dependent on question 5, option 1 "Yes")*

□ Patient satisfaction

□ Departmental pain management level

□ Nursing screening and assessment skills

□ Other _________________

30. After implementing the screening and assessment fee, what other aspects do you think need to be improved for better management of patient pain?

[Multiple-choice question]

*(Dependent on question 5, option 1 "Yes")*

□ Nursing screening and assessment ability

□ Optimization of communication and feedback between doctors and nurses

□ Attention to cancer pain

□ Analgesic treatment methods

□ Management of adverse reactions to analgesic drugs

□ Patient education

□ Other _________________

***General Questions***

31. What are the current methods of communication and feedback between doctors and nurses for cancer pain management in your department?

[Multiple-choice question]

□ Group handoff/shift report

□ Verbal communication

□ WeChat group communication

□ Pain handoff logbook

□ Other _________________

32. How many times have you participated in training related to cancer pain screening and assessment?

[Single-choice question]

○ Less than once a year

○ Once a year

○ Twice a year

○ 3 times a year

○ More than 3 times a year

33. What is your level of interest in training and experience sharing related to cancer pain screening and assessment?

[Single-choice question]

○ Very interested

○ Will participate if conditions permit

○ Generally do not participate

○ Not interested

34. What percentage of overall cancer pain management in your department do you consider satisfactory?

[Single-choice question]

○ Below 10%

○ 11%-25%

○ 26%-50%

○ 51%-75%

○ 76%-90%

○ Above 91%

35. Among the cancer pain patients treated in your department, what percentage of breakthrough pain management do you consider satisfactory?

[Single-choice question]

○ Below 10%

○ 11%-25%

○ 26%-50%

○ 51%-75%

○ 76%-90%

○ Above 91%

36. How long after the first onset of pain do patients generally seek medical attention?

[Single-choice question]

○ Less than 10 days

○ 11-30 days

○ 2-3 months

○ 4-6 months

○ 7-12 months

○ More than 13 months

○ Other _________________

37. What are the commonly used drugs for cancer pain management in your department? (You can select two or more)

[Multiple-choice question]

□ Oxycodone hydrochloride controlled-release tablets

□ Morphine sulfate controlled-release tablets

□ Morphine hydrochloride controlled-release tablets

□ Acetaminophen and oxycodone tablets

□ Acetaminophen and codeine tablets

□ Acetaminophen and dihydrocodeine tablets

□ Tramadol tablets/capsules

□ Bucinnazine (Qiangtongding tablets)

□ Bucinnazine (Qiangtongding injection)

□ Immediate-release morphine tablets

□ Morphine injection

□ Tramadol injection

□ Pethidine (Demerol)

□ Fentanyl transdermal patch

□ Celecoxib capsules (Celebrex)

□ Saridon (Propyphenazone/Paracetamol/Caffeine)

□ Ibuprofen tablets

□ Oxycodone hydrochloride immediate-release capsules

□ Other (Drug and dosage form) _________________
